# Elevated Peripheral Brain-Derived Neurotrophic Factor Level Associated With Decreasing Insulin Secretion May Forecast Memory Dysfunction in Patients With Long-Term Type 2 Diabetes

**DOI:** 10.3389/fphys.2021.686838

**Published:** 2022-01-17

**Authors:** Xi Huang, Zuolin Xie, Chenchen Wang, Shaohua Wang

**Affiliations:** ^1^Department of Endocrinology, Affiliated Zhongda Hospital of Southeast University, Nanjing, China; ^2^Nanjing Medical University, Nanjing, China; ^3^School of Medicine, Southeast University, Nanjing, China

**Keywords:** brain-derived neurotrophic factor, mild cognition impairment, type 2 diabetes, islet, insulin

## Abstract

**Background:**

With the progressive course of diabetes and the decline in islet function, the cognitive dysfunction of patients aggravated.

**Objective:**

We aimed to investigate the roles of brain-derived neurotrophic factor (BDNF) and the Val66Met polymorphism in mild cognitive impairment (MCI) in patients with type 2 diabetes mellitus (T2DM).

**Methods:**

A total of 169 Chinese patients with T2DM were involved and divided into long-term (diabetes duration >10 years) and short-term (diabetes duration ≤10 years) diabetes, and in each group, the patients were separated as MCI and the control. Demographic characteristics, clinical variables, and cognitive performances were assessed. The plasma BDNF level was measured *via* enzyme-linked immunosorbent assay. The Val66Met polymorphisms were analyzed.

**Results:**

Long-term T2DM have lower 2 h postprandial C-peptide (*p* < 0.05). The BDNF level was slightly higher in patients with MCI than in the controls in each duration group without statistical significance. The relationship of BDNF to Montreal Cognitive Assessment was not proven either. However, in the long-term diabetes group, BDNF concentration remained as an independent factor of logical memory test (β = −0.27; *p* < 0.05), and they were negatively correlated (*r* = −0.267; *p* = 0.022); BDNF was also negatively correlated with fasting C-peptide (*r* = −0.260; *p* = 0.022), 2 h postprandial C-peptide (*r* = −0.251; *p* = 0.028), and homeostasis model assessment of insulin resistance (*r* = −0.312; *p* = 0.006). In genotypic groups, BDNF Val/Val performed better in logical memory test than Met/Met and Val/Met.

**Conclusion:**

Elevated peripheral BDNF level associated with declined islet function, when combined with its Val66Met polymorphism, may forecast memory dysfunction in patients with long-term T2DM.

## Introduction

Brain-derived neurotrophic factor (BDNF) belongs to a family of neurotrophins that play a crucial role in the survival and differentiation of the neuronal population (Kowianski et al., 2018; von Bohlen Und Halbach and von Bohlen Und Halbach, 2018). BDNF is abundantly expressed in the central and peripheral nervous systems (Karczewska-Kupczewska et al., 2011), and it may play a non-substitutable role in the hippocampus in supporting the electrophysiological function of memory circuitry (Nagahara and Tuszynski, 2011). Evidence shows that BDNF can cross the blood-brain barrier in both directions (Pan et al., 1998; Karczewska-Kupczewska et al., 2011; Jiang et al., 2018); thus, the correlation between circulating BDNF and brain levels and brain function has been discussed in animal and human studies (Hwang et al., 2015; Weinstein et al., 2017; Ng et al., 2019).

Novel BDNF-focused investigations are being developed for both metabolism and neurological disorders. In neurodegenerative disorders (Weis et al., 2003; Blesch, 2006), including Alzheimer’s disease (AD; Lima Giacobbo et al., 2019; Ng et al., 2019) and mild cognitive impairment (MCI; Nagata et al., 2014), the levels of BDNF is one of the most common physiological changes. BDNF plays a critical role in cognition and memory (Kowianski et al., 2018; von Bohlen Und Halbach and von Bohlen Und Halbach, 2018; Miranda et al., 2019). Meanwhile, peripheral BDNF is observed in various metabolic conditions, including high cholesterol, insulin insensitivity, and type 2 diabetes mellitus (T2DM; Hölscher, 2011; Weinstein et al., 2017; Movassat et al., 2019). BDNF increases insulin sensitivity in the brain and periphery (Marosi and Mattson, 2014). Besides, the discovery of brain-specific insulin signaling deficiencies in the early stages of AD pathogenesis has led to the designation of AD as “type 3 diabetes” (de la Monte, 2012; Kandimalla et al., 2017). MCI is considered an intermediate stage between the expected cognitive decline in normal aging and AD (Eshkoor et al., 2015), where memory loss is the main symptom. Thus, the relationship between BDNF and insulin may be a novel research focus in investigating the underlying mechanisms of T2DM and MCI. To our knowledge, in long-term T2DM, islet function decreases, and cognitive dysfunction emerges simultaneously (Kandimalla et al., 2017). It is reported that insulin secretion decreases during the first 10 years after the diagnosis of T2DM (Mauvais-Jarvis et al., 2004). Taking different T2DM duration into account, we must determine whether peripheral BDNF interacts with insulin to influence the cognition decline in MCI patients with T2DM.

Although evidence suggests that BDNF plays a key role in the process of AD and T2DM, the role and association between BDNF polymorphism with these two diseases have been extensively investigated. Among them, a naturally occurring polymorphism in the human BDNF gene, Val66Met, was observed. This single-nucleotide polymorphism (SNP) possibly contributes to altered BDNF activity-dependent secretion (Egan et al., 2003). Several studies have implicated the association or otherwise of this polymorphism with MCI (Lim et al., 2021), AD (Shen et al., 2018), obesity, and T2DM risk (Akbarian et al., 2018), but the exact relationship remains controversial. We examined this SNP in different cognition groups to determine its role in the MCI of T2DM.

## Materials and Methods

### Subjects

We involved 169 hospitalized patients with T2DM (100 men and 69 women, aged 50–75 years) from the Department of Endocrinology of the Affiliated Zhongda Hospital of Southeast University between August 2015 and August 2017. All patients provided signed, informed consent to participate in the study, which was approved by the Research Ethics Committee of the Affiliated Zhongda Hospital of Southeast University. This study was registered in the Chinese Clinical Trial Registry. The registration number is ChiCTR-OCC-15006060.

The subjects were diagnosed with T2DM according to the World Health Organization [WHO] (1999) criteria (Alberti and Zimmet, 1998) and divided into two groups according to the duration of more than 10 years or not more than 10 years. Meanwhile, the patients with MCI satisfied the diagnostic criteria for MCI proposed by the MCI Working Group of the European Consortium on Alzheimer’s Disease in 2006: (1) Cognitive complaints from the patient or family of the patient; (2) A reported decline in the cognitive function relative to that in the past year by the patient or guardian of the patient [clinical dementia rating (CDR) score of 0.5]; (3) Cognitive disorders as evidenced by a clinical evaluation (impairment in memory or other cognitive domains); (4) Absence of major repercussions in the activities of daily living; and (5) Absence of dementia (based on the Diagnostic and Statistical Manual of Mental Disorders-IV criteria) (Portet et al., 2006). The exclusion criteria included severe hypoglycemia, diabetes ketoacidosis, hyperosmolar non-ketotic diabetic coma, acute cardiovascular or cerebrovascular accident, history of stroke (Hachinski ischemic score ≥4), head injury, alcoholism, Parkinson’s disease, epilepsy, major depression, other physical and mental illnesses, severe visual or hearing loss, and major medical illness (e.g., cancer, anemia, and serious infection).

### Clinical Data Collection

Demographic characteristics and medical history were collected by a member of the research staff. Physical measurement was observed in a standard way. Body mass index was calculated as body weight divided by the square of the height [body weight (kg)/body height (m^2^)]. Blood samples were drawn from the subjects the following morning after their admission to the hospital. C-peptide, triglyceride (TG), total cholesterol (TC), high-density lipoprotein (HDL-C), uric acid (UA), and thyroid-stimulating hormone (TSH) were tested using an automatic biochemical analyzer (Roche Group, Basel, Switzerland). Low-density lipoprotein (LDL-C), apolipoprotein A1 (ApoA1), apolipoprotein B (ApoB), and (lipoprotein a) Lpa were measured using an automatic biochemical analyzer (Ningbo Ruiyuan Biotechnology Co., Ltd., Ningbo, China). Glycosylated hemoglobin (HbA1c) was tested using an analyzer (Bio-Rad Laboratories, Inc., United States). Insulin resistance was calculated according to homeostasis model assessment of insulin resistance (HOMA-IR) using the formula for C-peptide: FPG (mmol/L) × FCP (nmol/L)/22.5 (Kim et al., 2016). The preceding biochemical measurements were performed in the central laboratory of the Affiliated Zhongda Hospital, which implemented internal and external quality control procedures directed by the Chinese Laboratory Quality Control.

### Neuropsychological Function Evaluation

Participants completed neuropsychological testing comprising the following.

#### Montreal Cognitive Assessment

Montreal Cognitive Assessment is a rapid sensitive assessment tool for screening mild cognition dysfunction, and the total score was 30 points (Nasreddine et al., 2005).

#### Mini-Mental State Examination

The examination was composed of 30 items, in which for each item, a correct answer is awarded one point, with a total of 30 points (Folstein et al., 1975).

#### Digit Span Test

The subjects were instructed to match a symbol with a number within 90 s according to the association between the number and the symbol.

#### Verbal Fluency Test

Verbal fluency test (VFT) assesses the correct number of animals, vegetables, fruits, and common grocery items identified within 1 min.

#### Clock Drawing Test

The participants were asked to draw the face of a clock.

#### Auditory Verbal Learning Test

Auditory verbal learning test (AVLT) evaluated a wide range of auditory and verbal learning functions. Immediate recall, short and long-delayed recall were recorded.

#### Logical Memory Test

Logical memory test (LMT) assessed immediate recall and delayed recall of a short story. The score was calculated as the total number of idea units recalled by the subjects.

#### Stroop Color-Word Test

Stroop Color-Word Test (SCWT) assessed the time taken to correctly identify the number of instances in which the color of the written word in identified (A time and A number), color of the symbol (B time and B number), and the color of the ink rather than the color the word spells (C time and C number).

#### Trail Making Test-A and Trail Making Test-B

Trail Making Test-A and Trail Making Test-B indicate the mean times taken to complete part A (visual conceptual) and part B (visuomotor tracking, which involves motor speed and attention functions).

Montreal Cognitive Assessment and MMSE scores were used to assess global cognitive abilities. SCWT, DST, VFT, and TMTB were conducted to evaluate the executive functions of the patients. CDT was used to analyze visual space function. TMTA test was performed to assess the information processing speed function. LMT and AVLT were used to analyze episodic memory.

Hachinski’s ischemic score, clinical dementia rating, the activity of daily living scale, and self-rating depression scale were obtained simultaneously. An experienced neurologist from the Department of Neurology in the Affiliated Zhongda Hospital of Southeast University performed all procedures, and the participants were blinded to the study design. The duration of the whole examination was approximately 45 min.

### Measurement of Plasma Brain-Derived Neurotrophic Factor Level

Blood samples were collected *via* venipuncture between 6:30 a.m. and 7:00 a.m. following an overnight fast. The samples were placed in anticoagulant tubes and centrifuged at 100 × *g* for 15 min to separate the plasma, which was stored at −80°C until analysis. Plasma BDNF concentrations were measured on the same day using enzyme-linked immunosorbent assay kits (Nanjing Jin Yibai Biological Technology Co., Ltd., Nanjing, China) per the instructions of the manufacturer. Intra- and inter-assay coefficients of variance were less than 9 and 10%, respectively, and the sensitivity limit of the assay was 0.5 μg/L.

### DNA Extraction and Single-Nucleotide Polymorphism Genotyping

Genomic DNA was extracted from the stored EDTA anticoagulated-venous blood using a DNA purification kit (Puregene, Gentra Systems, Minneapolis, MN, United States). Then, the genotype of BDNF rs6265 was detected using the Sequenom method (Beijing Cnkingbio Biotechnology Corporation, Beijing, China). The following forward and reverse primers were used: 5′-ACGTTGGATGTTGT TTTCTTCATTGGGCCG-3′ and 5′-ACGTTGGATGGCTT GACATCATTGGCTGAC-3′, respectively. The target region was amplified using PCR Accessory Set (Sequenom, 11327). The PCR products were processed using iPLEX Gold reagents and purified using a SpectroCHIP Resin kit (Sequenom, 10117-2). MassARRAY^®^ Analyzer (Agena, 260000) was used to acquire data from the purified samples. No inconsistency was found when 10% of the samples were randomly selected and genotyped again.

### Statistical Analysis

Data were presented as mean ± standard error of the mean (SEM), median (interquartile range), or percentage. Demographic, clinical characteristics, and neuropsychological test scores of the MCI and control patients were compared using Student’s *t*-test for normally distributed variables, the non-parametric Mann–Whitney *U*-test for asymmetrically distributed variables, and chi-squared (χ^2^) test for the qualitative variables. The test was also conducted to evaluate the distribution of genotypes and allele frequencies and to determine the deviations from the Hardy–Weinberg equilibrium (Santiago Rodriguez, Tom R. Gaunt, and Ian N. M. Day, Hardy–Weinberg Equilibrium Testing of Biological Ascertainment for Mendelian Randomization Studies). Analysis of variance was used to compare the plasma level of BDNF, glycosylated hemoglobin, and cognitive domains between different genotypes, and the least significant difference method was used for comparisons within each simple genotype group. The partial correlation analysis was performed to explore the relationships between BDNF and cognitive performances. Three hierarchical multiple regression models were used to estimate the predictor variables for LMT in patients with T2DM. In model 1, unique associations of the demographic factors to LMT were tested. Relational factors were added in model 2, and unique associations between health status covariates with LMT beyond effects of the demographic factors were tested. Finally, BDNF was added in model 3, and the unique associations between BDNF with LMT beyond effects of the demographic factors and health status covariates were tested. The linear correlation analysis was conducted to determine the relationship between BDNF and C-peptide, HOMA-IR. Statistical analyses were conducted using SPSS version 19.0 (SPSS Inc., Chicago, IL, United States).

## Results

### Demographic Characteristics, Clinical Characteristics, and Cognitive Performance

A total of 51 patients with MCI and 118 patients with healthy cognition were involved and divided into two groups according to the duration of T2DM. The demographic characteristics, clinical characteristics, and neuropsychological test scores of the participants are listed in Supplementary Table 1. Patients with T2DM for more than 10 years were older, have a longer hypertension duration, and lower 2 h postprandial C-peptide (2hCP) than those with T2DM for not more than 10 years (all *p* < 0.05). A comparison of the MCI group and the control in each diabetes duration group is shown in Table 1. The plasma BDNF level of MCI was slightly higher than that of the control, but the difference was not significant.

**TABLE 1:** Baseline characteristics of T2DM patients with different duration.

	Diabetes duration >10 years (*n* = 77)		Diabetes duration ≤10 years (*n* = 92)	
Characteristic	MCI group (*n* = 29)	Con group (*n* = 48)	MCI group (*n* = 22)	Con group (*n* = 70)
Age (y)	64.24 ± 8.24	61.58 ± 8.39	63.50 (49.75, 67.25)	55.5 (51.00, 63.00)
Male sex, n (%)	12.00 (41.40)	29.00 (60.40)	10.00 (45.50)*^c2^	49.00 (70.00)
Fatty liver, n (%)	16.00 (55.20)	20.00 (41.70)	15.00 (68.20)	37.00 (52.90)
Education level (y)	10.00 (9.00, 12.00)	11.00 (9.00, 12.00)	9.00 (8.00, 9.75)**^b2^	11.00 (9.00, 12.00)
Smoking, n (%)	8.00 (27.60)	17.00 (35.40)	2.00 (9.10)	26.00 (37.10)
Drinking, n (%)	3.00 (10.30)	11.00 (22.90)	2.00 (9.10)	17.00 (24.30)
Hypertension, n (%)	20.00 (69.00)	27.00 (56.30)	13.00 (59.10)	33.00 (47.10)
Hypertension duration (y)	8.00 (0.00, 15.50)	9.57 (0.00, 16.50)	3.00 (0.00, 8.25)	0.00 (0.00, 8.00)
Insulin use, n (%)	13.00 (44.80%)	33.00 (68.80)	11.00 (50.00)	45.00 (64.30)
Metformin use, n (%)	17.00 (58.60)	39.00 (81.30)	13.00 (59.10)	45.00 (66.20)
BMI (Kg/m^2^)	24.62 ± 3.17	25.00 ± 3.34	25.13 (23.44, 27.08)	24.47 (22.36, 26.44)
Weight (kg)	65.00 (58.00, 72.00)	70.00 (60.25, 76.00)	72.00 (60.00, 78.25)	69.00 (60.00, 80.00)
Height (cm)	163.00 (155.00, 169.00)*^b1^	168.00 (160.00, 172.00)	165.00 (158.00, 172.25)	169.00 (160.00, 175.00)
Systolic BP (mmHg)	132.00 (120.00, 150.00)	140.00 (122.25, 150.75)	134.50 (114.75, 140.00)	130.50 (123.50, 147.00)
Diastolic BP (mmHg)	80.00 (73.00, 87.00)	80.00 (74.00, 90.00)	80.00 (69.50, 86.00)	80.00 (74.75, 89.25)
HbA1c (%)	9.80 (7.65, 10.85)	8.20 (7.70, 9.38)	8.60 (7.93, 9.60)	8.65 (7.58, 10.33)
FCP (ng/mL)	0.47 (0.26, 0.64)	0.58 (0.32, 1.02)	0.69 (0.43, 0.88)	0.59 (0.37, 0.88)
2hCP (ng/mL)	1.63 (1.17, 2.35)	2.11 (1.21, 3.09)	2.10 (1.42, 3.11)	2.47 (1.61, 3.50)
HOMA-IR	0.06 (0.04, 0.09)	0.07 (0.03, 0.12)	0.09 (0.05, 0.15)	0.07 (0.04, 0.11)
TG (mmol/L)	1.49 (0.88, 1.94)	1.23 (0.87, 1.85)	1.89 (0.99, 2.26)	1.43 (1.00, 2.28)
TC (mmol/L)	4.69 ± 0.83	4.36 ± 1.20	4.62 ± 1.24	4.50 ± 1.03
HDL-C (mmol/L)	1.14 (1.03, 1.37)	1.08 (0.89, 1.42)	1.09 (0.92, 1.39)	1.05 (0.93, 1.28)
LDL-C (mmol/L)	2.89 ± 0.92	2.71 ± 0.85	2.86 ± 1.03	2.80 ± 0.83
ApoA1 (g/L)	1.16 (0.99, 1.37)	1.15 (0.92, 1.44)	1.20 ± 0.33*^a2^	1.06 ± 0.24
ApoB (g/L)	0.81 (0.69, 1.00)	0.76 (0.67, 0.96)	0.86 (0.65, 1.13)	0.81 (0.70, 0.95)
HDL-C/LDL-C	0.40 (0.33, 0.59)	0.44 (0.35, 0.52)	0.37 (0.32, 0.53)	0.40 (0.33, 0.51)
ApoA1/ApoB	1.41 (1.13, 1.95)	1.43 (1.17, 2.02)	1.28 (1.14, 1.76)	1.34 (1.06, 0.61)
LPa (mmol/L)	144.00 (71.50, 284.00)	143.50 (74.25, 240.75)	208.50 (127.25, 331.75)	134.00 (70.75, 269.75)
UA (μmol/L)	283.00 (246.50, 340.00)	292.50 (253.25, 337.75)	303.00 (233.75, 348.25)	306.50 (262.00, 358.75)
TSH (μIU/ml)	2.76 (1.93, 3.57)*^b1^	2.08 (1.22, 2.68)	2.43 (1.53, 4.23)*^b2^	1.81 (1.16, 2.57)
MOCA	24.00 (22.00, 24.00)**^b1^	28.00 (27.00, 29.00)	23.50 (22.00, 24.25)**^b2^	28.00 (27.00, 29.00)
MMSE	27.00 (26.00, 28.50)**^b1^	29.00 (29.00, 30.00)	27.00 (25.00, 28.00)**^b2^	29.00 (29.00, 30.00)
CDT	3.00 (2.50, 4.00)**^b1^	4.00 (3.25, 4.00)	3.00 (3.00, 4.00)*^b2^	4.00 (3.75, 4.00)
DST	11.00 (10.00, 12.00)*^b1^	12.00 (11.00, 13.00)	11.00 (9.75, 11.00)**^b2^	13.00 (11.00, 14.00)
VFT	15.00 (13.00, 17.00)**^b1^	18.00 (15.00, 21.00)	14.00 (13.00, 15.00)**^b2^	17.00 (15.00, 22.00)
TMTA	75.00 (57.00, 84.50)**^b1^	56.00 (48.00, 64.75)	68.50 (53.50, 83.75)*^b2^	54.00 (45.00, 65.00)
TMTB	189.00 (141.00, 265.50)**^b1^	131.50 (106.25, 172.00)	180.00 (156.50, 214.50)**^b2^	125.50 (95.00, 164.00)
SCWT A time	32.00 (25.50, 43.00)	28.50 (24.00, 35.75)	33.00 (25.00, 49.50)	28.50 (23.00, 35.50)
SCWT A number	50.00 (50.00, 50.00)	50.00 (50.00, 50.00)	50.00 (49.00, 50.00)**^b2^	50.00 (50.00, 50.00)
SCWT B time	59.00 (43.50, 67.50)*^b1^	44.50 (37.50, 55.00)	51.50 (39.25, 75.00)*^b2^	44.50 (35.00, 52.00)
SCWT B number	48.00 (47.00, 50.00)*^b1^	49.50 (48.00, 50.00)	48.50 (46.75, 50.00)**^b2^	50.00 (49.00, 50.00)
SCWT C time	100.00 (77.50, 125.50)	85.00 (71.75, 117.75)	116.00 (88.50, 138.25)**^b2^	76.00 (66.75, 89.25)
SCWT C number	44.00 (42.50, 48.00)**^b1^	48.00 (46.00, 49.00)	45.50 (42.00, 48.00)**^b2^	48.00 (47.00, 50.00)
AVLT immediate	16.03 ± 5.03*^a1^	19.23 ± 5.54	16.32 ± 4.51**^a2^	19.84 ± 4.72
AVLT delayed	4.66 ± 2.60**^a1^	6.56 ± 2.53	4.86 ± 1.88**^a2^	6.74 ± 2.63
LMT	6.93 ± 3.63**^a1^	11.13 ± 4.35	7.77 ± 3.72**^a2^	11.69 ± 4.20
BDNF (μg/L)	5.85 (4.88, 9.92)	5.35 (4.27, 8.30)	6.83 (4.88, 10.17)	5.71 (4.59, 8.47)

*Significance, p < 0.05; **Significance, p < 0.01.

*Data are presented as n (%), mean ± SD, or median (interquartile range) as appropriate. ^a1^Student’s t-test for the comparison of normally distributed quantitative variables between the MCI group and the control group in diabetic duration more than 10 years. ^a2^Student’s t-test for the comparison of normally distributed quantitative variables between the MCI group and control group in diabetic duration no more than 10 years. ^b1^Mann–Whitney U-test for the comparison of asymmetrically distributed quantitative variables between the MCI group and the control group in diabetic duration more than 10 years. ^b2^Mann–Whitney U-test for the comparison of asymmetrically distributed quantitative variables between the MCI group and control group in diabetic duration no more than 10 years. ^c1^Chi-square (χ^2^) test was used to compare qualitative variables between the MCI group and the control group in diabetic duration more than 10 years. ^c2^Chi-square (χ^2^) test was used to compare qualitative variables between the MCI group and the control group in diabetic duration no more than 10 years.*

*MCI, mild cognitive impairment; HbA1c, glycosylated hemoglobin; FPG, fasting plasma glucose; 2hPG, 2 h plasma glucose; FCP, fasting C-peptide; 2hCP, 2 h postprandial C-peptide; BMI, body mass index; WC, waist circumference; HC, hip circumference; WHR, waist-hip ratio; HOMA-IR, Homeostasis Model Assessment-Insulin Resistance; TG, triglyceride; TC, total cholesterol; LDL-C, low-density lipoprotein; HDL-C, high-density lipoprotein-C; ApoA1, apolipoprotein A1; ApoB, apolipoprotein B; MOCA, Montreal Cognitive Assessment; MMSE, Mini-Mental State Examination; CDT, Clock Drawing Test; DST, Digit Span Test; VFT, Verbal Fluency Test; TMTA, Trail Making Test-A; TMTB, Trail Making Test-B; AVLT, Auditory Verbal Learning Test; LMT, Logical Memory Test; SCWT, Stroop Color-Word Test; BDNF, brain-derived neurotrophic factor.*

### Relationship Between Brain-Derived Neurotrophic Factor and Cognitive Performances

The respective correlations between plasma BDNF level and cognitive performances were analyzed. When sex, age, TSH, and education levels were controlled, the partial correlation indicated that in the patients with T2DM for more than 10 years, BDNF was negatively related to LMT (*r* = −0.267; *p* = 0.022) (Table 2). The relationship of BDNF to other cognition performances was not proven.

**TABLE 2:** Partial correlation analysis between BDNF, clinical indicators, and LMT score of T2DM patients with different durations.

	LMT			
	
	Diabetes duration >10 years		Diabetes duration ≤10 years	
	*r*	*p*	*r*	*p*
BDNF (μg/L)	−0.267	0.022	−0.014	0.896
Metformin use	0.311	0.007	0.060	0.579
TC (mmol/L)	−0.301	0.009	0.029	0.792
HDL-C (mmol/L)	−0.286	0.014	−0.061	0.575

We performed the hierarchical multiple regression analysis in patients with T2DM for not more than 10 years, with LMT score as the dependent variable. The demographic factors, sex, age, and education levels, tested in model 1, did not explain meaningful variance in LMT scores. Health status covariates, total cholesterol, and high-density lipoprotein (HDL), which are tested in model 2, explain 11% of the variance in the LMT score beyond the effects of the demographic factors. In step 3, BDNF accounted for a significant proportion of the LMT score controlling beyond the effects of the demographic and health status covariates (Δ*R*^2^ = 0.07; *p* < 0.05). Additionally, in this final model, BDNF (β = −0.27; *p* < 0.05) was independently related to the LMT score, and the final model explained 23% of the variance in the LMT score (Table 3).

**TABLE 3:** Hierarchical multiple regression analysis of factors associated with LMT score in T2DM more than 10 years.

	Model 1		Model 2		Model 3	
Variables	Standardized β	95% CI	Standardized β	95% CI	Standardized β	95% CI
Age (year)	−0.10	−0.18 to 0.07	−0.09	−0.17 to 0.07	−0.08	−0.16 to 0.07
Sex	−0.17	−3.61 to 0.53	−0.20	−3.77 to −0.20	0.18	−3.58 to 0.25
Education level	0.07	−0.23 to 0.42	0.13	−0.14 to 0.50	0.15	−0.09 to 0.53
TC (mmol/L)			−0.21	−1.99 to 0.21	−0.25	−2.14 to 0.01
HDL-C (mmol/L)			−0.18	−5.68 to 1.06	−0.13	−5.03 to 1.54
BDNF (μg/L)					−0.27*	−0.87 to −0.10
ΔR^2^		0.05		0.11		0.07
ΔF		1.19		4.56*		6.26*

*p < 0.05.

### Relationship Between Brain-Derived Neurotrophic Factor to Insulin Secretion and Insulin Resistance

The linear correlation analysis was conducted between BDNF and indicators of islet secretion in subjects with diabetes for more than 10 years, which presented a negative relationship [FCP (*r* = −0.260; *p* = 0.022); 2hCP (*r* = −251; *p* = 0.028); HOMA-IR (*r* = −0.312, *p* = 0.006)]. In the group with T2DM for not more than 10 years, the correlation between BDNF and insulin secretion was not proven (Table 4).

**TABLE 4:** Relationship between BDNF and clinical indicators in T2DM patients with different durations.

	BDNF			
	DM duration >10 years		DM duration ≤10 years	
	*r*	*P*	*r*	*p*
FCP (ng/mL)	−0.260	0.022	−0.156	0.137
2hCP (ng/mL)	−0.251	0.028	−0.072	0.497
HOMA-IR	−0.312	0.006	−0.160	0.127

### Distributions of Brain-Derived Neurotrophic Factor Genotype and Allele Frequencies Between Groups

Distributions of BDNF genotype and allele frequencies of the MCI and the control subjects are presented in Table 5, which are consistent with the Hardy–Weinberg equilibrium in the MCI group (χ^2^ = 2.497, df = 2, *p* > 0.05) and control groups (χ^2^ = 0717, df = 2, *p* > 0.05). No significant differences were observed in the distributions between the two groups.

**TABLE 5:** Distributions of BDNF genotype and allele frequencies between groups.

		Genotype, n (%)				Allele, n (%)	

Group	Met/Met	Val/Val	Val/Met	*p*-value	Met	Val	*p*-value
MCI	18 (35.3)	11 (21.6)	22 (43.1)	0.111	58 (56.9)	44 (43.1)	0.149
Control	24 (20.3)	28 (23.7)	66 (55.9)		114 (48.3)	122 (51.7)	

*Data are presented as n (%).*

### Comparison of Plasma Brain-Derived Neurotrophic Factor Level, Logical Memory Test, Insulin Secretion, and Resistance Between Genotypic Subgroups

The differences in plasma BDNF level, insulin secretion, and resistance of BDNF rs6265 polymorphism groups are not significant as summarized in Table 6. However, the effect of genotype on the LMT scores was significant (*F* = 4.435, *p* = 0.013), while that of the Val/Val group is more remarkable than those of the Met/Met group (mean difference = 2.621, *p* = 0.008) and Val/Met (mean difference = 2.253, *p* = 0.009) (Table 6).

**TABLE 6:** Comparison of plasma BDNF level, LMT, insulin secretion, and resistance between genotypic subgroups.

	Met/Met (*n* = 42)	Val/Val (*n* = 39)	Val/Met (*n* = 88)	*p*
FCP (ng/mL)	0.69 (0.40, 1.03)	0.51 (0.29, 0.87)	0.56 (0.37, 0.84)	0.83
2hCP (ng/mL)	2.37 ± 1.21	2.23 ± 1.75	2.41 ± 1.30	0.93
HOMA-IR	0.07 (0.05, 1.12)	0.05 (0.04, 0.10)	0.07 (0.04, 0.11)	0.87
BDNF (μg/L)	5.97 (4.78, 9.57)	6.47 (4.78, 9.45)	5.37 (4.42, 7.84)	0.138
LMT	10.00 (5.00, 13.00)	12.00 (9.00, 15.00)	10.00 (7.00, 13.00)	0.013

*Data are presented as mean ± SEM, or median (interquartile range) as appropriate.*

## Discussion

In this observational study, we first compared the plasma level of 2hCP in patients with >10 years and ≤10 years of T2DM and found it was obviously lower in the group of long-term duration. Second, this study compared the plasma levels of BDNF in patients with T2DM between groups with or without MCI in the different diabetes duration groups. In each group, compared with control, the BDNF level of the MCI group was slightly higher, but the difference was not significant. Third, we conducted a correlation analysis between BDNF and cognitive performance (e.g., MMSE, MOCA, LMT, and other neuropsychological tests) and only found a significant relationship between LMT and BDNF. Only in the individuals with T2DM for more than 10 years, the plasma BDNF levels were obviously negatively correlated with the LMT scores, which was iterated *via* hierarchical multiple regression. Fourth, the plasma BDNF was negatively associated with FCP, 2hCP, and HOMA-IR only in the longer duration group. Finally, in terms of polymorphism, the Val homozygote genotype performed best in the LMT test.

In this study, long-term T2DM has a lower 2hCP. This result corresponds with several studies, which reports that insulin secretion decreases in long-term T2DM either (Zangeneh et al., 2006). The difference in BDNF levels between the MCI and control groups was not significant, which was consistent with a meta-analysis of 2019 on the serum BDNF level in MCI (Ng et al., 2019). Higher, lower, or unchanged results have been described in patients with AD compared with healthy controls, but the reasons for such different findings are unclear (Balietti et al., 2018). According to the classification of neuropsychological assessment proposed by van den Berg et al. (2009), the cognitive domains that should be evaluated include memory, intelligence, processing speed, and attention. BDNF promotes synaptic plasticity and is crucial for hippocampus-dependent learning and memory (Binder and Scharfman, 2004). Thus, single cognition domains, such as LMT and testing logic memory, should be discussed specifically. In our examination, the negative correlation between peripheral BDNF level and LMT score was presented among patients with T2DM who were diagnosed for more than 10 years, which is a new supplement about the circulation BDNF levels of patients with AD. We hypothesize that on early cognition dysfunction, damaged logic memory might be the trigger of the compensatory increase in BDNF. This result was found only in the group of patients with long-term T2DM. The different insulin secretion between long and short diabetes duration might be the reason, which was observed in our study either.

Meanwhile, the correlation between BDNF and insulin in different diabetes duration was analyzed. To date, increased (Suwa et al., 2006; Boyuk et al., 2014) and decreased (Krabbe et al., 2007) BDNF were reported in the blood of patients with DM. Our results indicated that, especially in the group with long-term diabetes, the serum BDNF level showed a negative correlation with FCP, 2hCP, and HOMA-IR, and this result is consistent with that of a study of Chinese patients with T2DM (Li et al., 2016). Those results demonstrated that the plasma BDNF level can be regulated only when insulin secretion was decreasing and might explain the ability of BDNF to compensate for cognition decline in long-term diabetes duration.

In terms of genetic factors, to date, the effect of BDNF Val66Met polymorphism on cognition decline remains controversial. The Val allele may be neuroprotective and related to higher cognition, and Met is related to impaired cognitive function (Azeredo et al., 2017; Barha et al., 2019). A recent meta-analysis indicated that performance on memory-based tasks was better in Val/Val carriers (Toh et al., 2018), which is partly consistent with our results. Moreover, it was observed that Met was associated with greater memory decline by using an episodic memory composite score over 126 months, while the effect of BDNF on memory decline was proved to be greatest in preclinical AD. However, in the MCI group, there was no effect of Met66 on memory decline or disease progression to AD over 126 months (Lim et al., 2021). Similarly, in our study, no significant differences were noted between genotypic subgroups in either the MCI or control group. But LMT scores of Val/Val carriers were significantly higher than other patients, which was used to assess the episodic memory performance of patients. Besides, the role of the BDNF Val66Met SNP in episodic memory was also reported in other studies (Cathomas et al., 2010; Kennedy et al., 2015). These results support our hypothesis that on early cognition dysfunction, damaged memory might be correlated with BDNF. In our results, the BDNF Val66Met SNP did not affect plasma BDNF levels, which is consistent with other findings. It did not affect the FCP, 2hCP, and HOMA-IR of T2DM, although Val/Val is prone to developing T2DM (Daily and Park, 2017).

However, several limitations should be considered in our study. First, the relatively small sample size limited the interpretation of our results. Second, the course of disease development was not studied, and thus, a prospective study should be conducted. However, this study is one of the first to investigate the relationship between plasma BDNF, insulin, cognition domain in different duration of T2DM, and the BDNF rs6265 polymorphism. In summary, we suggest that the plasma BDNF in long diabetic duration, correlated with insulin secretion, might contribute to the compensatory rise in memory deficit. BDNF might be the early screening indicator of memory deficit in long-term T2DM before AD was defined. Our results also support a role for the Val66Met BDNF polymorphism in episodic memory. Given that either AD or MCI is a complex disorder, the overall susceptibility cannot be fully explained by diabetes. The specificity of the correlation between Val66Met polymorphisms and the respective cognition domains should be explored further as it may provide insights into the development and progression of various subtypes of AD and MCI.

## Data Availability Statement

The raw data supporting the conclusions of this article will be made available by the authors, without undue reservation.

## Author Contributions

SW: study concept, design, and interpretation. XH: writing, analysis, and interpretation. ZX and CW: acquisition of data and study supervision. All authors contributed to the article and approved the submitted version.

## Conflict of Interest

The authors declare that the research was conducted in the absence of any commercial or financial relationships that could be construed as a potential conflict of interest.

## Publisher’s Note

All claims expressed in this article are solely those of the authors and do not necessarily represent those of their affiliated organizations, or those of the publisher, the editors and the reviewers. Any product that may be evaluated in this article, or claim that may be made by its manufacturer, is not guaranteed or endorsed by the publisher.
