# Supplementary material for: Elevated Peripheral Brain-Derived Neurotrophic Factor Level Associated With Decreasing Insulin Secretion May Forecast Memory Dysfunction in Patients With Long-Term Type 2 Diabetes
Source: Front Physiol. 2022 Jan 17;12:686838. doi: 10.3389/fphys.2021.686838 (PMC8801615; doi:10.3389/fphys.2021.686838)
Supplement: Supplementary file 1 [file Table_1.DOCX]

Supplementary Material

Supplementary Table 1. Baseline demographic, clinical characteristics and cognitive performances in study subjects.

| characteristic | Total(n=169) | Diabetes duration＞10y (n=77) | Diabetes duration≤10y (n=92) | P value |
| --- | --- | --- | --- | --- |
| Age (y) | 59.85±9.07 | 68.58±8.38 | 57.57±9.03 | <0.0001 |
| Male sex, n (%) | 100(59.17) | 41(53.25) | 59.00(64.13) | 0.152 |
| Fatty liver, n (%) | 88.00(52.07) | 36.00(46.75) | 52.00(56.52) | 0.454 |
| Education level (y) | 10.00(9.00,12.00) | 9.00(10.00,12.00) | 10.00(9.00,12.00) | 0.986 |
| Smoking, n (%) | 53.00 (31.36) | 25.00(32.47) | 28.00(30.43) | 0.777 |
| Drinking, n (%) | 33.00(19.52) | 14.0(18.18) | 19.00(20.65) | 0.687 |
| Hypertension, n (%) | 93.00(55.03) | 47.00(61.04) | 46.00(50.00） | 0.151 |
| Hypertension duration (y) | 2.00(0.00,10.50) | 9.13(0.00,15.50) | 5.00(0.00,8.00) | 0.005 |
| Insulin use, n (%) | 101.00(59.76) | 46.00(59.74) | 55.00(59.78) | 0.927 |
| Metformin use, n (%) | 114.00(68.26) | 56.00(72.72) | 58.00(64.44) | 0.252 |
| BMI | 24.60(22.72,26.72) | 24.49(22.65,26.70) | 24.62±3.17 | 0.893 |
| Weight (kg) | 67.00(60.00,76.00) | 65.60(59.50,75.00) | 70.00(60.00,79.00) | 0.269 |
| Height (cm) | 167.00(158.00,172.00) | 165.00(158.00,171.50) | 168.50(160.00,175.00) | 0.105 |
| SystolicBP (mmHg) | 135.00(122.00,150.00) | 139.00(121.00,150.00) | 131.50(122.25,145.75) | 0.348 |
| DiastolicBP (mmHg) | 80.00(73.50,89.00) | 80.00(74.00,89.50) | 80.00(72.00,88.75) | 0.927 |
| HbA1c (%) | 8.60(7.70,10.10) | 8.50(7.70,10.25) | 8.60(7.63,9.90) | 0.979 |
| FCP (ng/mL) | 0.57(0.37,0.88) | 0.56(0.32,0.93) | 0.60(0.39,0.88) | 0.490 |
| 2hCP (ng/mL) | 2.11(1.42,3.15) | 1.92(1.22,2.72) | 2.22(1.61,3.47) | 0.015 |
| HOMA-IR | 0.07(0.04,0.11) | 0.06(0.04,0.11) | 0.07(0.04,0.11) | 0.308 |
| TG (mmol/L) | 1.40(0.94,2.14) | 1.27(0.88,1.89) | 1.43(1.00,2.26) | 0.056 |
| TC (mmol/L) | 4.51±1.08 | 4.49±1.08 | 4.53±1.08 | 0.787 |
| HDL (mmol/L) | 1.09(0.94,1.34) | 1.10(0.97,1.39) | 1.07(0.92,1.30) | 0.187 |
| LDL (mmol/L) | 2.80±0.87 | 2.78±0.87 | 2.82±0.88 | 0.774 |
| ApoA1 (g/L) | 1.12(0.94,1.32) | 1.15(0.95,1.41) | 1.08(0.94,1.26) | 0.040 |
| ApoB (g/L) | 0.80(0.68,0.95) | 0.77(0.68,0.97) | 0.82(0.69,0.95) | 0.691 |
| HDL/LDL | 0.40(0.33,0.52) | 0.42(0.34,0.53) | 0.39(0.33,0.51) | 0.159 |
| ApoA1/ApoB | 1.35(1.13,1.74) | 1.41(1.17,1.99) | 1.29(1.07,1.61) | 0.087 |
| LPa (mmol/L) | 148.00(73.00,271.50) | 144.00(73.00,245.50) | 150.00(73.00,294.50) | 0.853 |
| UA（umol/L） | 299.25(250.08,346.00) | 287.00(249.58,339.0) | 306.50(250.50,356.50) | 0.341 |
| TSH（uIU/ml） | 2.08(1.34,2.99) | 2.17(1.47,3.03) | 1.89(1.22,2.90) | 0.238 |
| MOCA | 27.00(25.00,28.50) | 27.00(24.00,28.00) | 27.00(26.00,29.00) | 0.155 |
| MMSE | 29.00(28.00,30.00) | 29.00(27.00,29.50) | 29.00(28.00,30.00) | 0.058 |
| CDT | 4.00(3.00,4.00) | 4.00(3.00,4.00) | 4.00(3.00,4.00) | 0.193 |
| DST | 12.00(11.00,13.00) | 12.00(11.00,13.00) | 12.00(12.00,14.00) | 0.298 |
| VFT | 16.00(14.00,19.50) | 17.00(15.00,19.00) | 16.00(14.00,19.75) | 0.398 |
| TMTA | 57.00(48.00,73.00) | 59.00(51.50,76.50) | 55.50(47.00,71.50) | 0.094 |
| TMTB | 141.00(107.50,192.50) | 157.00(115.00,196.00) | 134.50(99.00,183.50) **^**b1^** | 0.095 |
| SCWT A time | 30.00(24.00,37.00) | 30.00(24.00,36.00) | 30.00(23.00,38.00) | 0.985 |
| SCWT A number | 50.00(50.00,50.00) | 50.00(50.00,50.00) | 50.00(50.00,50.00) | 0.471 |
| SCWT B time | 46.00(37.50,60.00) | 46.00(41.00,62.00) | 45.50(37.00,56.75) | 0.182 |
| SCWT B number | 46.00(37.50,60.00) | 49.00(48.00,50.00) | 50.00(49.00,50.00) | 0.073 |
| SCWT C time | 85.00(71.00,116.50) | 92.00(74.00,120.00) | 82.00(69.00,101.25) | 0.027 |
| SCWT C number | 47.00(45.00,49.00) | 47.00(44.00,48.00) | 48.00(46.00,50.00) | 0.033 |
| AVLT immediate | 18.56±5.20 | 18.03±5.54**a | 19.00±4.89 | 0.226 |
| AVLT delayed | 6.09±2.64 | 5.84±2.70 | 6.29±2.59 | 0.272 |
| LMT | 10.20±4.50 | 9.55±4.56 | 10.75±4.40 | 0.083 |
| BDNF (ug/L) | 5.78(4.61,8.75) | 5.66(4.44,8.75) | 5.96(4.77,8.80) | 0.362 |

Data are presented as n (%), mean± SD, or median (interquartile range) as appropriate.

Abbreviations: MCI, mild cognitive impairment; HbA1c, glycosylated hemoglobin; FPG, fasting plasma glucose; 2hPG,2 hours plasma glucose; FCP, fasting C-peptide; 2hCP, 2 hours postprandial C-peptide; BMI, body mass index; WC, waist circumference; HC, hip circumference; WHR, waist-hip ratio; HOMA-IR, Homeostasis model assessment-insulin resistance; TG, triglyceride; TC, total cholesterol; LDL, low density lipoprotein; HDL, high density lipoprotein; ApoA1, apolipoprotein A1; ApoB, apolipoprotein B; MOCA, Montreal Cognitive Assessment; MMSE, Mini-mental State Examination; CDT, Clock Drawing Test; DST, Digit Span Test; VFT, Verbal Fluency Test; TMTA, Trail Making Test-A; TMTB, Trail Making Test-B; AVLT, Auditory Verbal Learning Test; LMT, Logical Memory Test; SCWT, Stroop Color Word Test; BDNF, brain derived neurotrophic factor.
